# Multidimensional spatial monitoring of open pit mine dust dispersion by unmanned aerial vehicle

**DOI:** 10.1038/s41598-023-33714-x

**Published:** 2023-04-26

**Authors:** Lin Li, Ruixin Zhang, Quansheng Li, Kai Zhang, Zhigao Liu, Zhicheng Ren

**Affiliations:** 1 China Energy Investment Group Co., Ltd, Beijing, 100011 China; 2 State Key Laboratory of Water Resource Protection and Utilization in Coal Mining, Beijing, 102209 China; 3 School of Energy and Mining, China University of Mining and Technology (Beijing), Beijing, 100083 China; 4 School of Computer Science, North China University of Science and Technology, Sanhe, 065201 China

**Keywords:** Environmental sciences, Engineering

## Abstract

Dust pollution is one of the most severe environmental issues in open pit mines, hindering green mining development. Open pit mine dust has characteristics of multiple dust-generating points, is irregular, influenced by climatic conditions, and has a high degree of distribution with a wide dispersion range in three dimensions. Consequently, evaluating the quantity of dust dispersion and controlling environmental pollution are crucial for supporting green mining. In this paper, dust monitoring above the open pit mine was carried out with an unmanned aerial vehicle (UAV) on board. The dust distribution patterns above the open pit mine were studied in different vertical and horizontal directions at different heights. The results show that the temperature changes less in the morning and more at noon in winter. At the same time, the isothermal layer becomes thinner and thinner as the temperature rises, which makes it easy for dust to spread. The horizontal dust is mainly concentrated at 1300 and 1550 elevations. The dust concentration is polarized at 1350–1450 elevation. The most serious exceedance is at 1400 elevation, with TSP (the concentration of total suspended particulate), PM10 (particulates with aerodynamic diameter < 10 μm), and PM2.5 (particulates with aerodynamic diameter < 2.5 μm) accounting for 188.8%, 139.5%, and 113.8%, respectively. The height is 1350–1450 elevation. Dust monitoring technology carried out by UAV can be applied to the study of dust distribution in the mining field, and the research results can provide reference for other open pit mines. It can also provide a basis for law enforcement part to carry out law enforcement, which has expanded and wide practical application value.

## Introduction

The open pit mining industry is an important basic industry related to China's economic lifeline and energy security, China's open pit coal production has been maintaining good growth momentum. The national open pit coal production reached 720 million t and 950 million t in 2016 and 2021, respectively. The total amount of open pit coal mining has far exceeded the United States, Australia, and other developed countries in the world^1–3^. However, in the process of open pit mining, productive dust occurs to varying degrees. With the emphasis on ecological civilization in China, environmental protection departments and social organizations have become less tolerant of productive dust from open pit mines and dust accumulation around open pit mines, causing some interference in the normalization of open pit mine production management and even forming a negative impact on the socially restrictive development of open pit mines^4^. Therefore, it is important to conduct research on the extent and dispersion pattern of dust dispersion in mining areas and open pit mine.

And the dust in open pit mine has strong specificity, which leads to the difficulty of original basic data collection. At present, dust monitoring for mines and open pit mines is mainly based on fixed ground monitoring^5–7^. Wanjun Tang et al.^8^ analyzed the dust transport pattern of open pit mines and the influencing factors, etc., through on-site monitoring in open pit mines. Wenqi Lu et al.^9^ used ground monitoring for dust monitoring, which illustrated the consistent pattern of PM2.5 and PM10 concentration changes. Luo Huaiting^10^ builtd a dust monitoring platform for analyzing the dust distribution pattern of open pit mines in winter. However, the dust in open pit mines is generally characterized by “many dust-producing points, high dispersion, and three-dimensional diffusion”, but the monitoring of open pit mines is mainly conducted by ground-based fixed-point monitoring, and the dust accumulation height and distribution range above the open pit mines cannot be known.

With the development of UAV monitoring technology, gradually move from ground monitoring to UAV monitoring to conduct pollution studies. Niu Ji et al.^11^ developed a six-rotor UAV dust monitoring system, which collected complete PM2.5 concentration data through UAV flight measurements. There was no significant change in the magnitude of PM2.5 concentration in the 0–150 m vertical direction in Nanchang area from the end of May to the beginning of June 2014. Yang Hai et al.^12^ implemented UAV for atmospheric environment monitoring using unmanned piggyback cameras and dust monitoring equipment. The ground verification and the analysis of the pollution gas concentration monitoring results at 150, 200, 250 and 350 m heights in the experimental area show that the pollution gas emissions in the chemical park have spatial horizontal differences and vertical dispersion characteristics. In 2015 Miguel Alvarado et al.^13^ integrated photoelectric dust sensors to a fixed-wing UAV for validating the feasibility of UAV-mounted sensors, further enriching the UAV monitoring approach. Tommaso Francesco Villa et al.^14^ adopted UAV-mounted monitoring equipment for air quality monitoring. The optimal mounting point of the sensor next to the UAV was determined, and the UAV propellers produced a dispersion effect that manifested itself in reduced gas and PN concentrations measured in real time. In 2017, Guo Wei et al.^15^ used a UAV with monitoring equipment in order to study the vertical distribution and dispersion pattern of PM2.5. 2017, Villa et al.^16^ used a UAV for vertical height pollutant monitoring. It demonstrates the impact of traffic emissions on human exposure, but less so to pollution within the upper part of the boundary layer. Ding Chengjun et al.^17^ designed a dust monitoring system based on UAV, this further increases the precision and accuracy of the monitoring system. Sławomir Pochwała et al.^18^ developed a UAV-based air quality monitoring system to identify the most dangerous areas. In the same year, Abhishek Anand et al.^19^ based on UAV technology targeted the monitoring of SO_2_, NO_2_, etc., enriching the field of UAV monitoring concentrations while ships were moving, and the reliability of the system was verified by simulations. The research of scholars at that time mainly applied the application of UAV monitoring to pollutant monitoring in urban and marine areas, which illustrated the feasibility and reliability of monitoring technology through UAVs. At present, there are few studies on dust monitoring in open pit mine using UAV technology, and the above technology applications provide technical support to carry out dust monitoring in open pit mine.

This paper takes China Coal Pingshuo Group Limited Anjialing Open Pit Mine (Hereinafter referred to as “the Anjialing open pit mine”) as the research object, and uses UAV monitoring technology to carry out research on dust monitoring at different heights above the open pit mine, establishes dust distribution models, studies dust concentration changes and distribution laws in vertical height and horizontal direction, and finally proposes targeted dust reduction measures according to dust distribution laws. This study has a certain reference value for monitoring dust concentration and taking dust reduction and dust suppression measures in environmental research and mining engineering fields.

## Materials and methods

**Figure 1:**
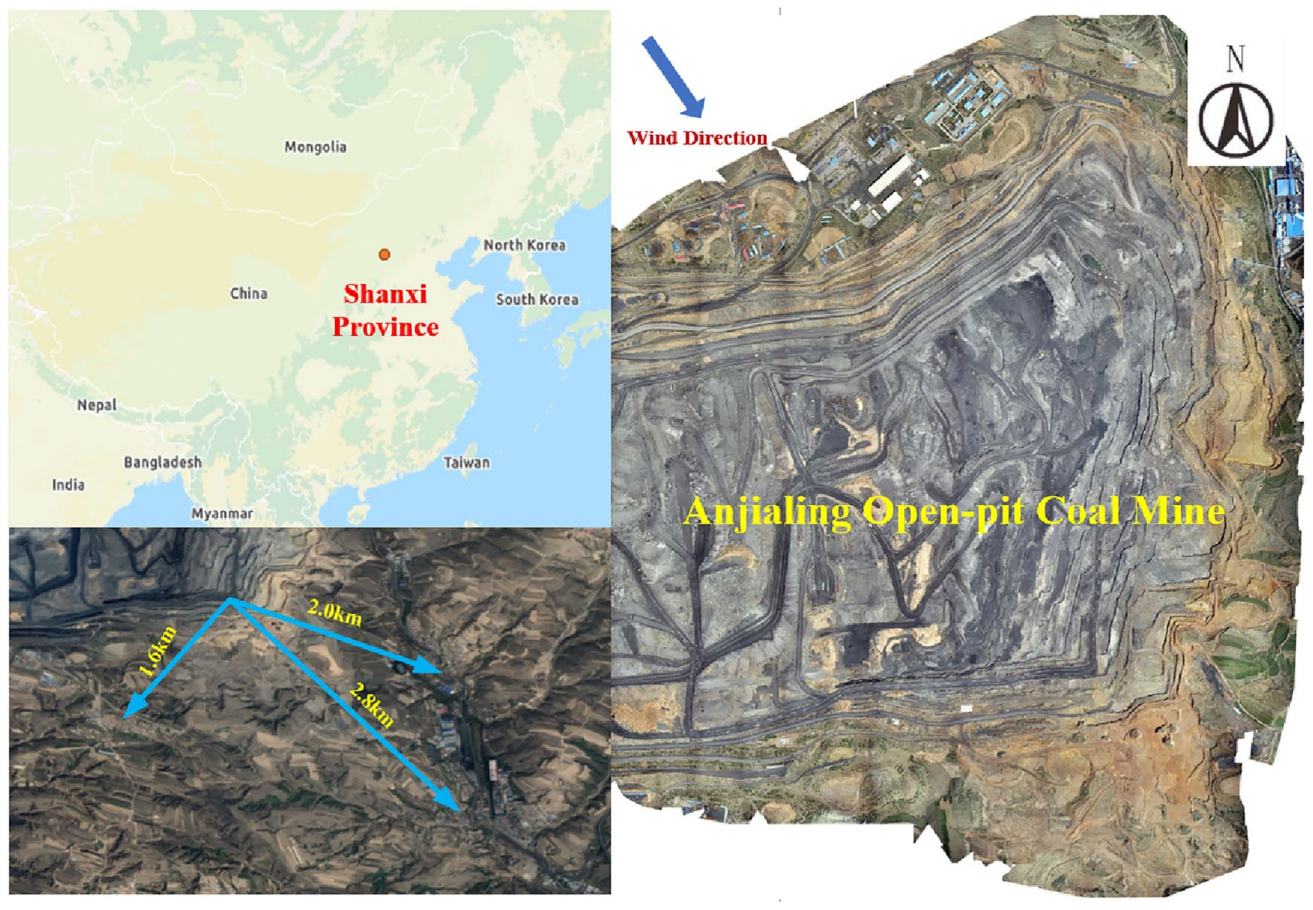
Location map of the study area. Maps were created using WPS for Windows (version 11.1.0.13703. https://www.wps.cn), the original image freely available in Amap (https://www.amap.com).

The Pingshuo mining area is located in the Pinglu District of Shuozhou City, Shanxi Province, China. It is one of the five largest open pit mining areas in China. The Anjialing open pit mine (located in Xiyi Village, Baitang Township, Pinglu District) is the second largest open pit mine in the Pingshuo mining area, with an annual production of 20 Mt/year and an average annual stripped rock volume of about 90 million m^3^/year. Three nearby villages are located 1.6 km, 2.0 km, and 2.8 km away from the Anjialing open pit mine. If the Anjialing open pit mine is environmentally polluted, it will inevitably affect the three villages, and the location of the study area is shown in Fig. 1. Therefore, it is very necessary to predict the dust concentration of the Anjialing open pit mine in Pingshuo, so that reasonable control measures can be taken in time.

### UAV monitoring program

Due to the limitation of traditional research means, it is difficult to solve the observation problem of near-open pit dust migration patterns and obtain the dust dispersion pattern at different levels and heights. The UAV has the characteristics of flexibility and mobility and controllable flight altitude, etc. By carrying sensors on the UAV, the three-dimensional and real-time dust concentration monitoring of the whole pit range can be carried out. Parameters such as PM2.5, PM10, and TSP concentrations at different seasons and altitudes can be obtained so that the quantitative research of dust dispersion laws can be realized.

**Figure 2:**
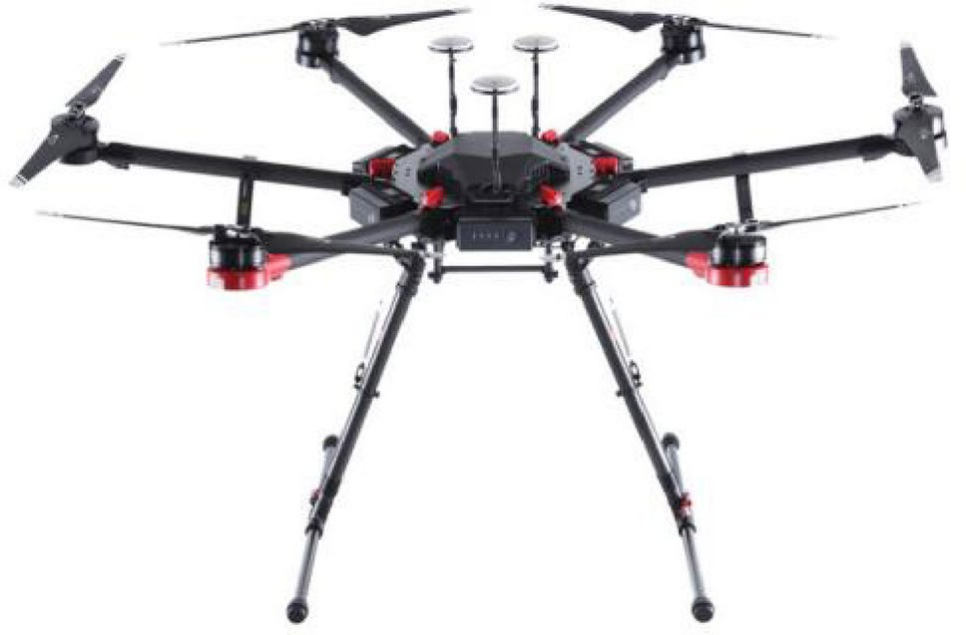
DJI M600pro UAV.

UAV platforms are mainly divided into fixed-wing UAVs^20^ and multi-rotor UAVs^21^ etc. While fixed-wing UAVs fly at high altitudes and are not conducive to near-surface dust monitoring, multi-rotor UAVs can take off and land vertically and have the advantage of being at lower altitudes and easier to operate than fixed-wing aircraft^22^, so multi-rotor UAVs are chosen to carry out dust monitoring. DJI UAVs have the advantages of good performance, safety, and stability. Therefore, DJI M600pro UAV is chosen for the study in this paper, as shown in Fig. 2.

DJI M600pro parameters are shown in Table 1.

**Table 1:** Parameters of DJI M600pro UAV.

Parameters	Size
Number of axes	6
Wheelbase	1133 mm
UAV weight	5.3 kg
Maximum takeoff weight	15.1 kg
Maximum ascent speed	5 m/s
Maximum descent speed	3 m/s
Hovering accuracy	Vertical: ± 0.5 m; Horizontal: ± 1.5 m
Maximum horizontal flight speed	18 m/s (in static wind environment)
Maximum flight altitude	500 m
Maximum withstand wind speed	8 m/s
Working environment temperature	− 10 to 40 °C

The monitoring equipment was selected as Sniffer4D, and the reliability of the sensor was tested by the authority of The South China National Metrology Test Center^23^. The Sniffer4D monitors at a frequency of 1 s, a resolution of 5 m, and a weight of 1 kg, with the parameters shown in Table 2.

**Table 2:** Monitoring instrument module parameters.

Parameters	Monitoring parameters	Measurement range	Resolution	Precision
Dust concentration monitoring	PM2.5	0–2000 μg/m^3^	0.01 μg/m^3^	± 10%
	PM10	0–2000 μg/m^3^	0.01 μg/m^3^	± 10%
	TSP	0–30,000 μg/m^3^	0.1 μg/m^3^	± 10%
Meteorological parameter monitoring	Temperature	− 40 to 80 °C	0.15 °C	± 0.1 °C
	Relative humidity	0–100%	0.1%	± 2%
	Air pressure	1–110 kpa	0.01 kpa	± 0.2 kPa

Considering the airflow disturbance between individual rotor blades in the multi-rotor system, the airflow disturbance caused by the rotor blades was simulated by Fluent software when considering the effect of air viscosity^24^. The results show that the air pressure above the center of the UAV is less disturbed by airflow, so installing the monitoring equipment above the UAV not only reduces the effect of UAV rotor vibration, but also ensures that the error of monitoring data is minimized. Therefore, the monitor was installed to the position with the least disturbance from the UAV according to the simulation results.

**Figure 3:**
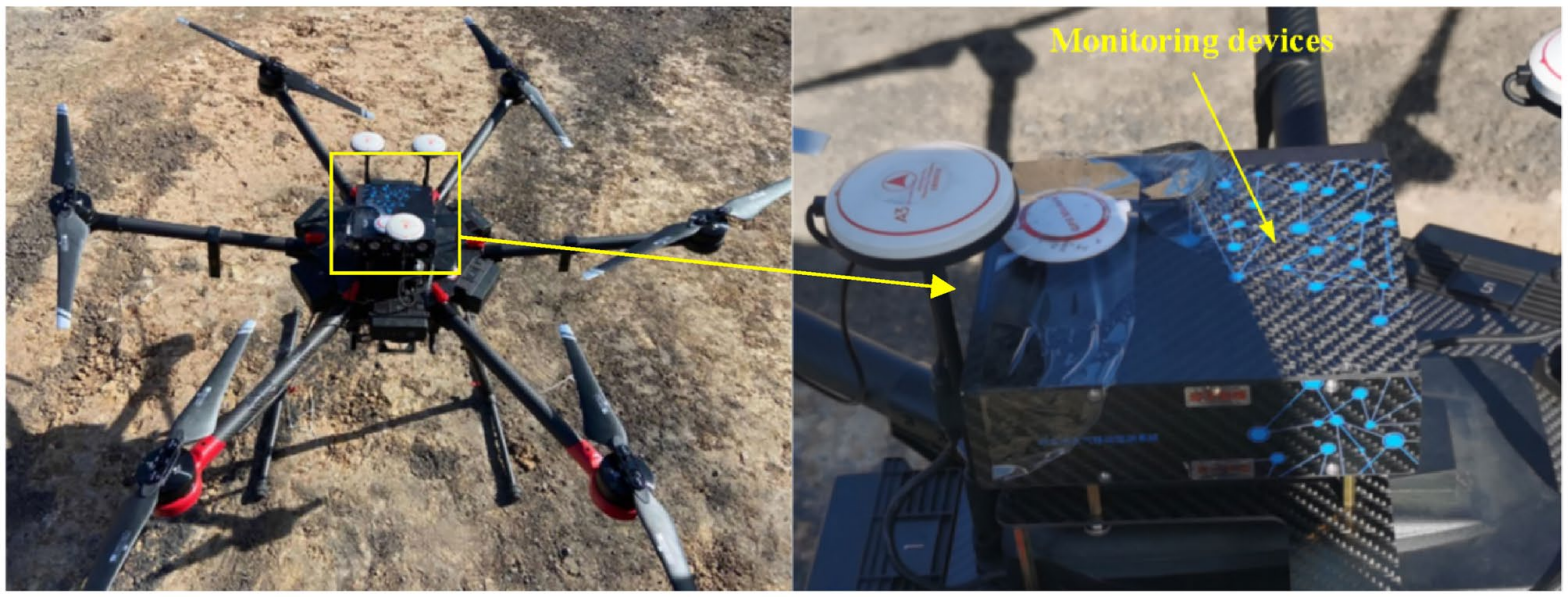
DJI M600pro with monitoring equipment.

The DJI M600pro is selected to carry the monitoring equipment in this study, as shown in Fig. 3.

**Figure 4:**
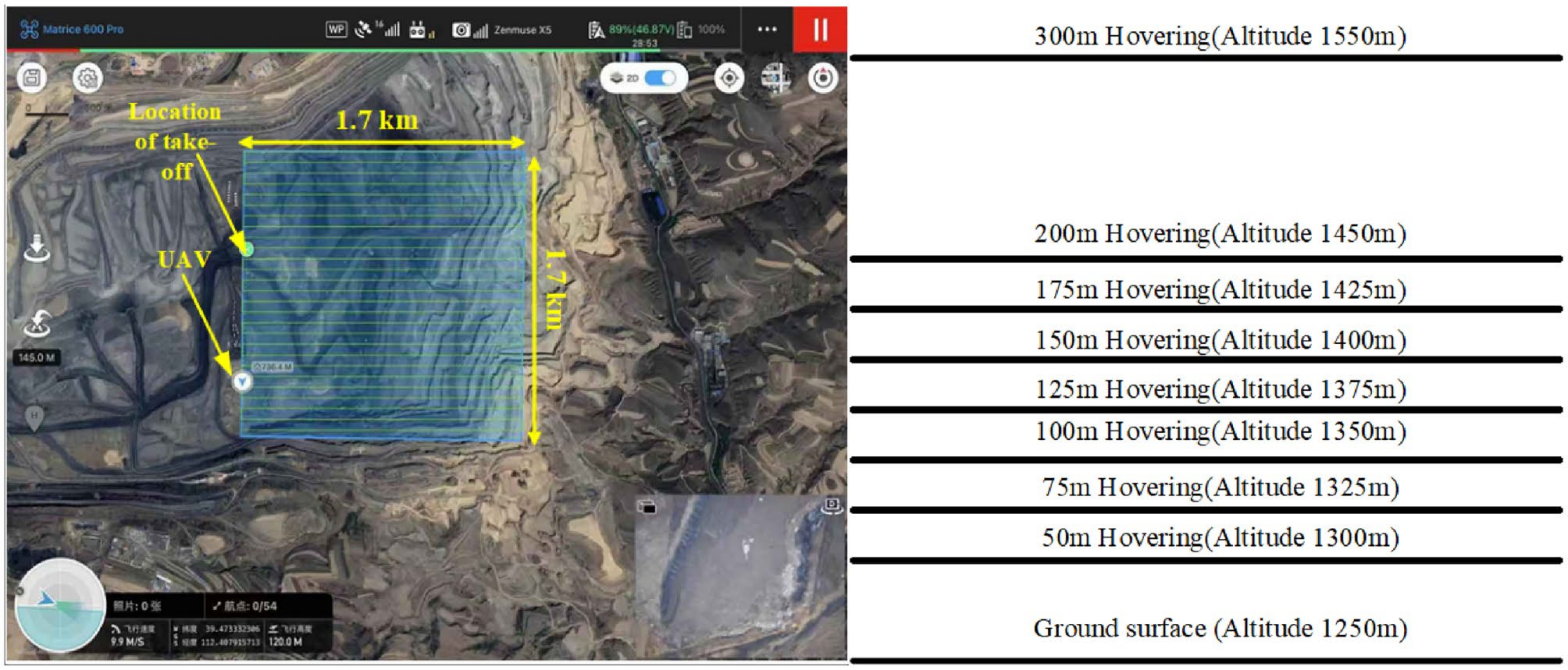
UAV route planning. Maps were created using WPS for Windows (version 11.1.0.13703. https://www.wps.cn), the original image freely available in Amap (https://www.amap.com).

The small observation platform at the elevation of 1250 in the Anjialing open pit mine was taken as the relative zero point, mainly because the fixed monitoring in the pit was mainly below 1250, and the UAV monitoring was used as a supplementary monitoring means to the ground monitoring. The dust concentration is collected at heights of 50 m, 75 m, 100 m, 125 m, 150 m, 175 m, 200 m, and 300 m above, respectively, and the horizontal flight area is chosen to be 1.7 × 1.7 km, which basically covers the open pit mine quarry, due to the limitations of the UAV range and safety. The UAV route planning is shown in Fig. 4.

### Monitoring data acquisition and pre-processing

The winter weather conditions at the mine site are shown in Table 3.

**Table 3:** Monitoring meteorological conditions.

Time	Weather conditions	Temperatures	Wind power wind direction
November 25, 2020	Overcast	− 1 °C/− 8 °C	Southeastern wind 1-2
November 26, 2020	Clear	1 °C/− 14 °C	West wind 1-2
November 27, 2020	Clear	− 2 °C/− 13 °C	Northwest wind 1-2
November 28, 2020	Clear	− 1 °C/− 12 °C	West wind 1-2
November 29, 2020	Clear	− 2 °C/− 13 °C	Northwest wind 1-2
November 30, 2020	Cloudy	− 4 °C/− 12 °C	Southeastern wind 1-2
December 1, 2020	Snow	− 1 °C/− 8 °C	Southeastern wind 1-2
December 2, 2020	Cloudy	− 5 °C/− 10 °C	Northwest wind 3-4
December 3, 2020	Clear	− 5 °C/− 17 °C	South wind 1-2

As can be seen from the table, except for December 2, 2020, the weather conditions tended to be stable, and the wind was mainly maintained at level 1–2, which was favorable to carry out on-site monitoring. At the same time, since the on-site meteorological conditions could not be controlled, we mainly referred to the ground meteorological conditions and chose to carry out monitoring during windless and breezy hours to ensure consistent external climatic conditions for monitoring as much as possible and reduce the influence brought by external meteorological conditions.

The number of sampling points at different elevations is shown in Table 4.

**Table 4:** Number of sampling points at different elevations in winter.

Data collection time	Height/m	Sampling points/pc
2020.11.25-12.3	1300	5742
	1325	4708
	1350	5003
	1375	5247
	1400	4670
	1425	5201
	1450	3994
	1550	12,771

As the UAV carries monitoring equipment for data collection process, it needs to power on and preheat the monitoring setup in advance. Therefore, when the data is output after the monitoring work is completed, the data needs to be pre-processed. The data from the preheating of the equipment is sieved, and the data volume consistency processing is performed to facilitate data analysis and ensure the reliability of the data. The valid data volumes of PM2.5, PM10, TSP, temperature, relative humidity and air pressure at different elevations after data volume consistency processing are shown in Table 5.

**Table 5:** Effective data volume at different elevations in winter.

Data collection time	Height/m	PM2.5/pc	PM10/pc	TSP/pc	Temperature/pc	Relative humidity/pc	Air pressure/pc
2020.11.25-12.3	1300	5596	5596	5596	5596	5596	5596
	1325	4060	4060	4060	4060	4060	4060
	1350	3691	3691	3691	3691	3691	3691
	1375	4947	4947	4947	4947	4947	4947
	1400	3776	3776	3776	3776	3776	3776
	1425	4103	4103	4103	4103	4103	4103
	1450	3791	3791	3791	3791	3791	3791
	1550	12,483	12,483	12,483	12,483	12,483	12,483

## Data analysis methods

UAV monitoring data are dust concentrations at different monitoring points at the same height, and spatial valuation is required to obtain dust concentrations in the same height plane. There are many kinds of spatial interpolation algorithms. The main ones commonly used are the inverse ratio interpolation algorithm and the kriging interpolation algorithm^25,26^. The kriging interpolation algorithm makes maximum use of the information provided by spatial sampling, considering not only the data of the sample point but also the data of the neighboring sample points, considering not only the spatial location of the sample point to be estimated and the neighboring known sample points, but also the location relationship between the neighboring sample points, and using the structural characteristics of the spatial distribution of the existing observations. Thus, the kriging interpolation algorithm is more accurate than other methods and can give an estimation error.kriging's estimation formula is.

1 $$Z^{*} \left( {x_{0} } \right) = \sum\limits_{i = 1}^{n} {\lambda_{i} } Z\left( {x_{i} } \right)$$

where $Z^{*} \left( {x_{0} } \right)$ is the estimated value at $x_{0}$; $Z\left( {x_{i} } \right)$ is the measured value at $x_{i}$.

The variance of the error in their estimates is:

2 $$S = 2\sum\limits_{i = 1}^{n} {\lambda_{i} \gamma \left( {x_{i} - x_{0} } \right)} - \sum\limits_{i = 1}^{n} {\sum\limits_{j = 1}^{n} {\lambda_{i} \lambda_{j} } } \gamma \left( {x_{i} - x_{j} } \right)$$

3 $$\sum\limits_{i = 1}^{n} {\lambda_{i} } = 1$$

4 $$\gamma \left( h \right) = \frac{1}{{2N_{h} }}\sum\limits_{i = 1}^{{N_{h} }} {\left[ {Z\left( {x_{i} + h} \right) - Z\left( {x_{i} } \right)} \right]}^{2}$$

where $\gamma \left( h \right)$ is the variance function and *h* is the separation distance; *N*_*h*_ is the logarithm of the samples used to calculate the sample variance function values between 0 $\left( {x_{i} + h,\,x_{i} } \right)$.

A Lagrange multiplier *μ* needs to be introduced in order to find the minimum value of the estimated variance.

5 $$S = \sum\limits_{i = 1}^{n} {\lambda_{i} } \gamma (x_{i} - x_{0} ) - {\kern 1pt} \sum\limits_{i = 1}^{n} {\sum\limits_{j = 1}^{n} {\lambda_{i} \lambda_{j} \gamma } } (x_{i} - x_{j} ) - 2\mu \left( {\sum\limits_{i = 1}^{n} {\lambda_{i} - 1} } \right)$$

To minimize the estimated variance, we have.

6 $$\left. \begin{gathered} \frac{\partial S}{{\partial \lambda_{1} }} = 0 \hfill \\ \frac{\partial S}{{\partial \lambda_{2} }} = 0 \hfill \\ \cdots \hfill \\ \frac{\partial S}{{\partial \lambda_{n} }} = 0 \hfill \\ \end{gathered} \right\}$$

According to the following equation.

7 $$\left. \begin{gathered} \sum\limits_{i = 1}^{n} {\lambda_{i} \gamma \left( {x_{i} - x_{j} } \right) + \mu = \gamma \left( {x_{j} - x_{0} } \right)} \hfill \\ \sum\limits_{i = 1}^{n} {\lambda_{i} = 1} \hfill \\ \end{gathered} \right\}$$

Namely.

8 $$\left| \begin{gathered} \gamma_{11} \cdots \gamma_{1n} {\kern 1pt} {\kern 1pt} {\kern 1pt} {\kern 1pt} {\kern 1pt} {\kern 1pt} {\kern 1pt} 1 \hfill \\ {\kern 1pt} {\kern 1pt} {\kern 1pt} {\kern 1pt} {\kern 1pt} \vdots {\kern 1pt} {\kern 1pt} {\kern 1pt} {\kern 1pt} {\kern 1pt} {\kern 1pt} {\kern 1pt} {\kern 1pt} {\kern 1pt} {\kern 1pt} {\kern 1pt} {\kern 1pt} {\kern 1pt} {\kern 1pt} {\kern 1pt} {\kern 1pt} {\kern 1pt} {\kern 1pt} {\kern 1pt} {\kern 1pt} \vdots {\kern 1pt} {\kern 1pt} {\kern 1pt} {\kern 1pt} {\kern 1pt} {\kern 1pt} {\kern 1pt} {\kern 1pt} {\kern 1pt} {\kern 1pt} {\kern 1pt} {\kern 1pt} {\kern 1pt} \vdots \hfill \\ \gamma_{n1} \cdots \gamma_{nn} {\kern 1pt} {\kern 1pt} {\kern 1pt} {\kern 1pt} {\kern 1pt} {\kern 1pt} 1 \hfill \\ 1{\kern 1pt} {\kern 1pt} {\kern 1pt} {\kern 1pt} {\kern 1pt} {\kern 1pt} {\kern 1pt} {\kern 1pt} {\kern 1pt} \cdots {\kern 1pt} {\kern 1pt} {\kern 1pt} {\kern 1pt} 1{\kern 1pt} {\kern 1pt} {\kern 1pt} {\kern 1pt} {\kern 1pt} {\kern 1pt} {\kern 1pt} {\kern 1pt} {\kern 1pt} {\kern 1pt} {\kern 1pt} {\kern 1pt} {\kern 1pt} 0 \hfill \\ \end{gathered} \right|{\kern 1pt} {\kern 1pt} {\kern 1pt} \left| \begin{gathered} {\kern 1pt} \lambda_{1} \hfill \\ {\kern 1pt} {\kern 1pt} {\kern 1pt} \vdots \hfill \\ {\kern 1pt} \lambda_{n} \hfill \\ {\kern 1pt} {\kern 1pt} \mu \hfill \\ \end{gathered} \right| = \left| \begin{gathered} {\kern 1pt} \gamma_{01} \hfill \\ {\kern 1pt} {\kern 1pt} {\kern 1pt} \vdots \hfill \\ {\kern 1pt} \gamma_{0n} \hfill \\ {\kern 1pt} {\kern 1pt} 1 \hfill \\ \end{gathered} \right|$$

All weights $\lambda_{1} , \ldots ,\lambda_{n}$ and Lagrange multipliers *µ* are obtained, where $\gamma_{ij} = \gamma (x_{i} - x_{j} )$ , i.e., the distance is the value of the variance function between *x*_*i*_ and *x*_*j.*_

Using the calculated weights and Lagrange multipliers, the estimated value Z* and the estimated variance can be obtained from Eq. (9).

9 $$\sigma^{2} = \sum\limits_{i = 1}^{n} {\lambda_{i} \gamma \left( {x_{0} - x_{i} } \right)} + \mu$$

## Analysis of results

### Analysis of the variation pattern of dust concentration at a vertical height

**Figure 5:**
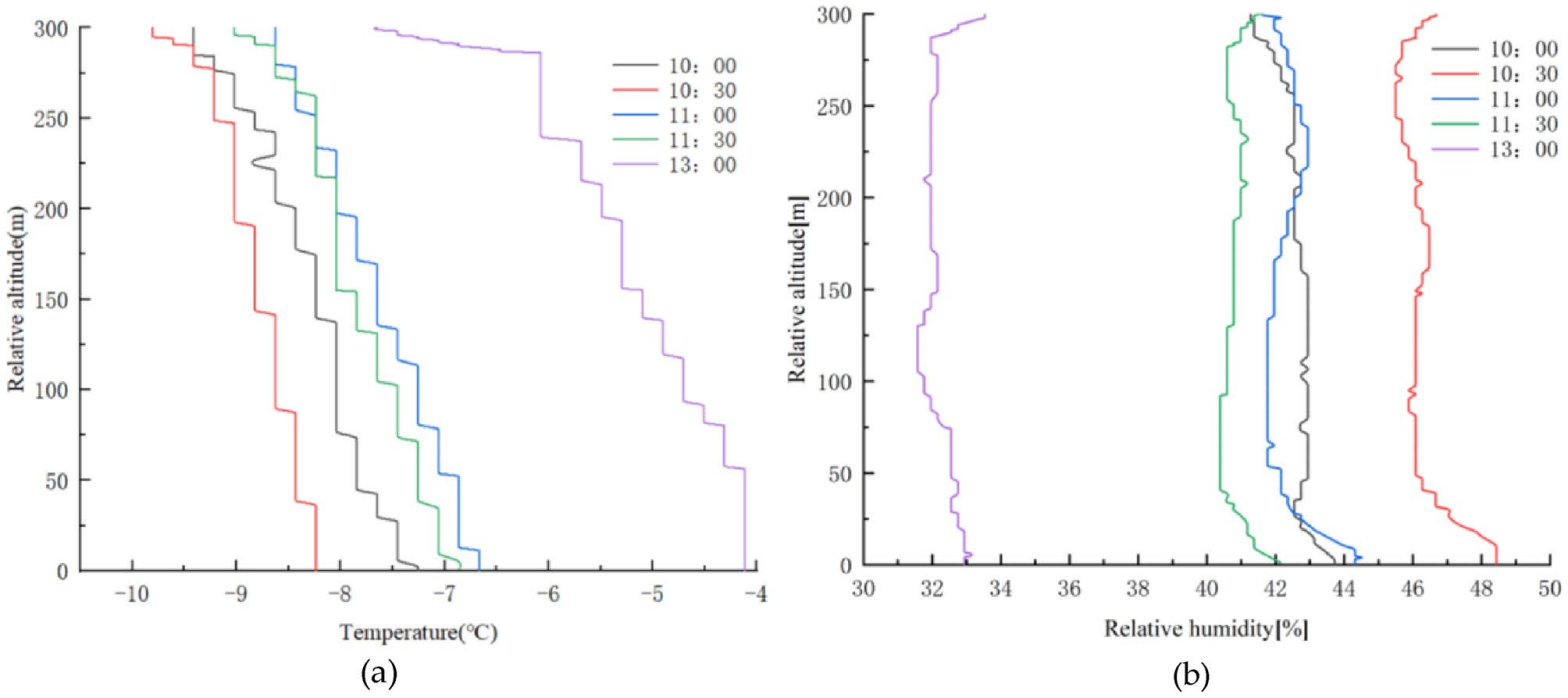
Temperature, relative humidity, and altitude relationship in winter; (**a**) Temperature-altitude relationship; (**b**) Relative humidity-altitude relationship.

The relationship between temperature, relative humidity, and altitude at the moment of 10:00 a.m.–13:00 p.m. is shown in Fig. 5.

From Fig. 5a, it can be seen that the temperature gradually increases with time and decreases with increasing height. There is a strong inverse temperature layer at the height of 225 m at 10:00 a.m. The vertical decreasing rate of temperature at 11:00 a.m. is the smallest at − 0.46 °C/100 m and the vertical decreasing rate at 13:00 p.m. is the largest at − 1.18 °C/100 m, indicating that while the isothermal layer is getting thinner and thinner as the temperature increases, which is constantly destroyed. From Fig. 5b, it can be seen that the relative humidity gradually decreases with time, 0–50 m relative humidity continuously decreases with the increase of height, at 50–200 m height, 10:30 a.m., 11:00 a.m., and 11:30 a.m. relative humidity increases with the increase of height, 46.8%, 42.9%, and 40.8%, respectively. 10:00 a.m. and 13:00 p.m. relative humidity changes are small, 200–300 m in height, with decreasing relative humidity at 10:00 a.m. and 11:00 a.m. and increasing relative humidity at 11:30 a.m. and 13:00 p.m.. At 13:00 p.m., the sunlight is at the strongest time of the day, so the temperature rises more, which leads to a high evaporation of water vapor in the air and a decrease in humidity. It indicates that the relative humidity is high and varies greatly in the morning and with the increase of temperature, the relative humidity decreases and varies little at noon, while the relative humidity is between 40% and 50%, which is not conducive to dust diffusion and deposition, causing dust accumulation in the morning, further explaining the phenomenon of high dust concentration in the morning and low dust concentration at noon.

**Figure 6:**
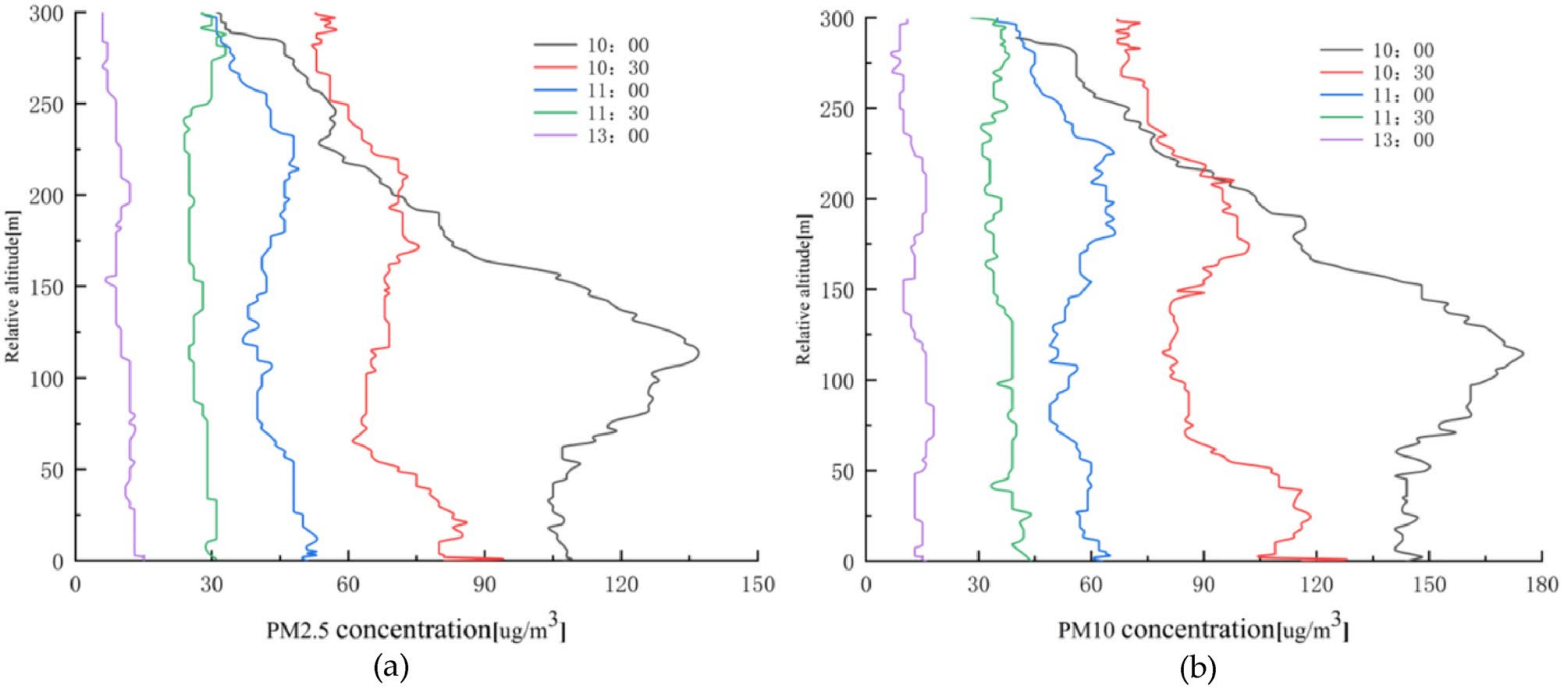
PM2.5 and PM10 concentrations versus height in winter; (**a**) PM2.5 concentration versus height; (**b**) PM10 concentration versus height.

The relationship between PM2.5 and PM10 concentrations and height is shown in Fig. 6.

From Fig. 6, it can be seen that PM2.5 and PM10 change in a similar pattern, and their concentrations keep decreasing with time. At 10:00 a.m., the dust concentration increases sharply to 137 μg/m^3^ and 175 μg/m^3^ at 0–100 m height, and then decreases to 32 μg/m^3^ and 35 μg/m^3^ with increasing height, and decreases at 0–75 m height at 10:30–11:00 a.m.. The dust concentration decreases continuously within the height of 0–75 m at 10:30–11:00 a.m., but the rate of decrease is the fastest at 10:30 a.m., and the dust concentration increases continuously within the height of 75–200 m. The decrease of dust concentration tends to be stable within the height of 200–300 m, but the rate of increase is lower than the rate of decrease of dust concentration from 0 to 75 m. The PM2.5 and PM10 concentrations change less with the temperature increase at 11:30 a.m. and 13:00 p.m., and the range of change is between 24–31 μg/m^3^, 24–31 μg/m^3^, 33–44 μg/m^3^ and 6–15 μg/m^3^, 7–18 μg/m^3^. This indicates that the PM2.5 and PM10 concentrations are high in the morning with a fast vertical decreasing rate and low at noon with little change in dust concentration in the vertical direction.

**Figure 7:**
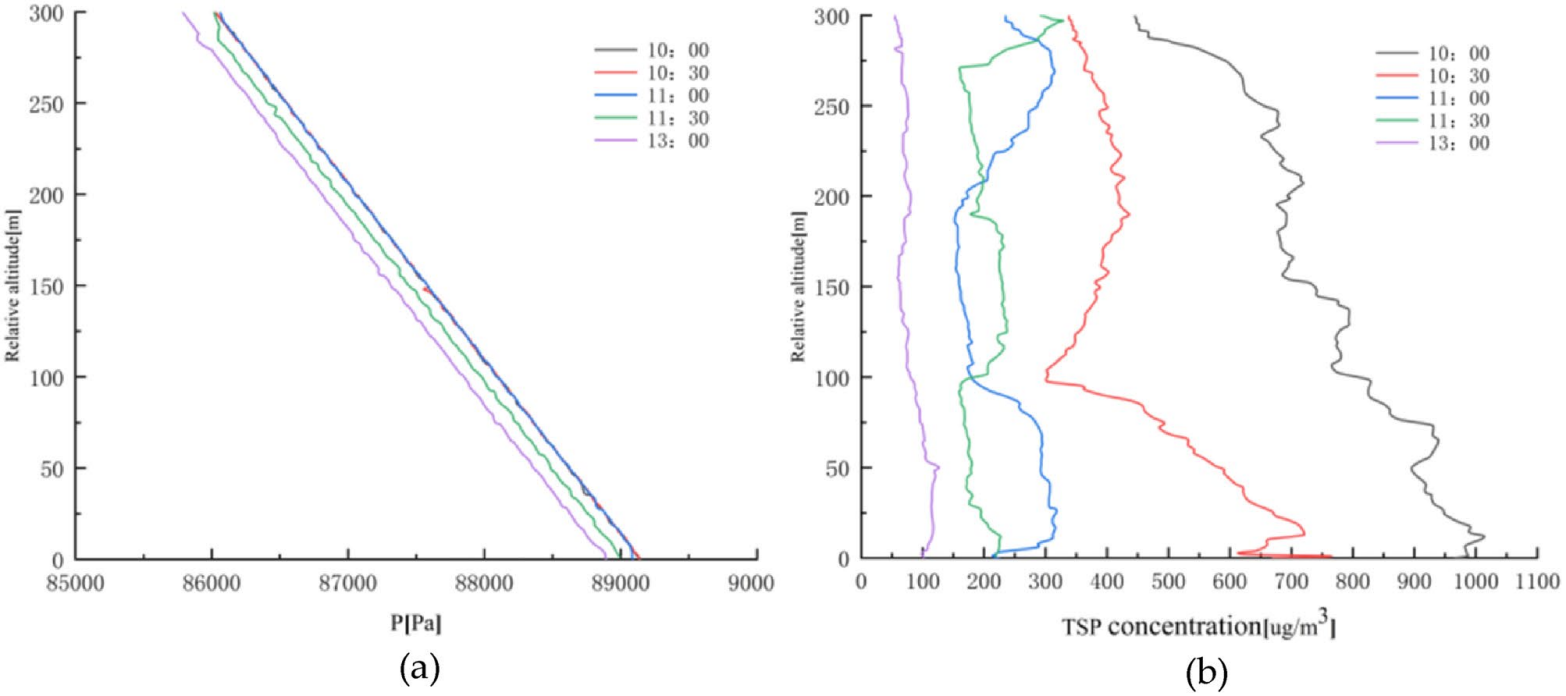
Relationship between air pressure, TSP concentration, and altitude in winter; (**a**) Air pressure versus altitude; (**b**) TSP concentration versus altitude.

The plot of air pressure, TSP, and altitude according to the monitoring data is shown in Fig. 7.

From Fig. 7a, it can be seen that the air pressure decreases continuously, mainly with increasing altitude, with the fastest decrease from 11:30 a.m. to 13:00 p.m.. There are sudden changes in air pressure at local places while decreasing continuously with time. It can be seen from Fig. 7b that TSP decreases continuously with time. At 10:00 a.m., the TSP concentration decreases continuously, but the local concentration increases at 50 m, 125 m, 200 m, and 250 m. At 10:30 a.m., the TSP concentration gradually decreases from 622 μg/m^3^ at 0–100 m to 301 μg/m^3^ and increases from 100–200 m to 436 μg/m^3^. The concentration of TSP at 200–300 m tended to be stable in the range of 338–360 μg/m^3^, with little change and stable distribution at 13:00 p.m.. This indicates that the TSP concentration is high in the morning. The vertical decreasing rate is fast, while the concentration is low at noon. The change of TSP concentration in the height direction is small.

### Analysis of the variation pattern of horizontal height dust concentration

**Figure 8:**
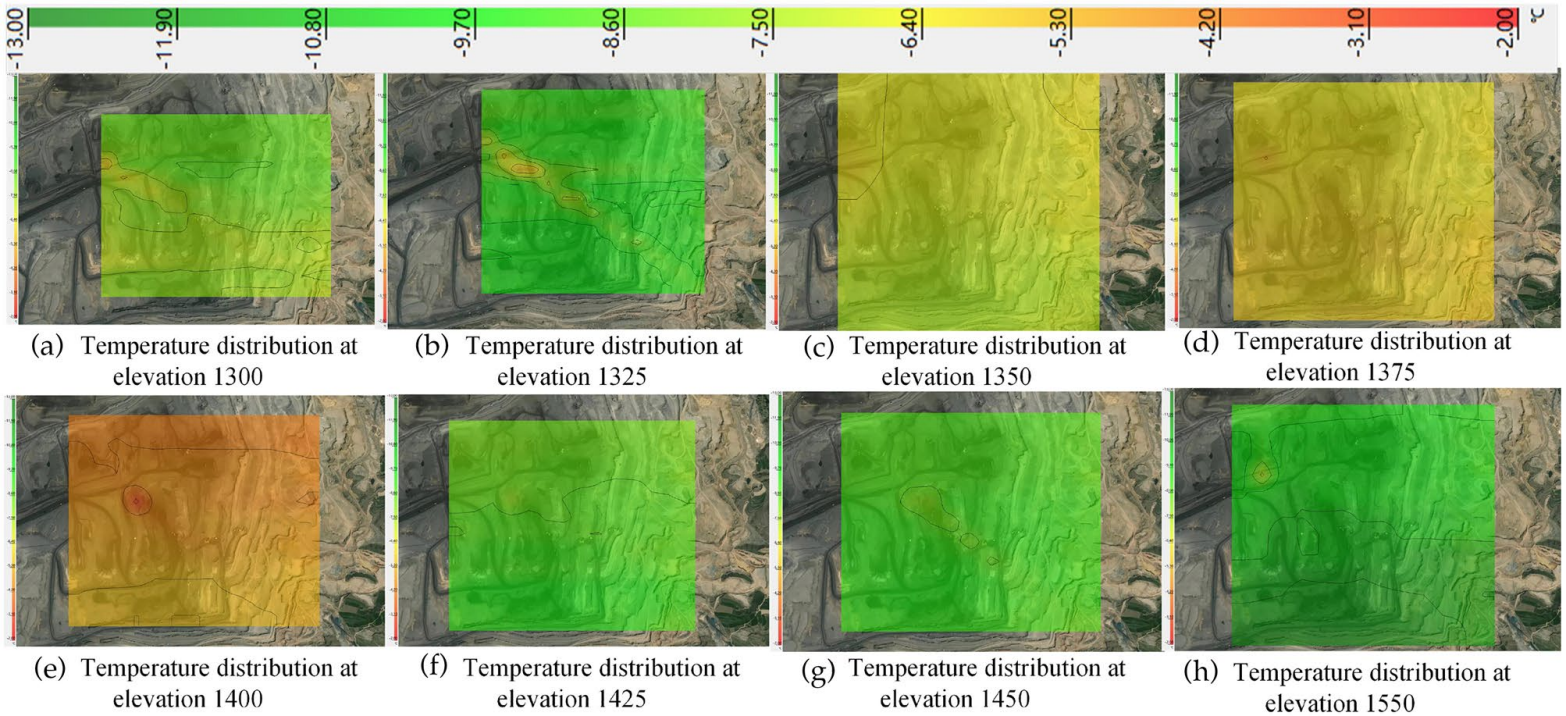
Temperature distribution from 1300 to 1550 elevation in winter.

Using the temperature − 13 °C to − 2 °C as the threshold, the temperature distribution from 1300 elevation (50 m height from the surface of the monitoring location) to 1550 elevation (300 m height from the surface of the monitoring location) was obtained by the kriging valuation method as shown in Fig. 8.

From Fig. 8, it can be seen that the temperature of the north gang is lower than that of the south gang at elevation 1300, and the temperature difference is 1 °C; at elevation 1325, the north is higher than that of the south; from elevation 1350–1425, the overall temperature is higher, which hinders the flow of air from below to above; from elevation 1450–1550, the north gang is higher than that of the south gang, and the temperature difference between the north and south at elevation 1550 is higher than that at elevation 1450. It indicates that the inverse temperature layer is mainly concentrated in the elevations from 1350 to 1425.

**Figure 9:**
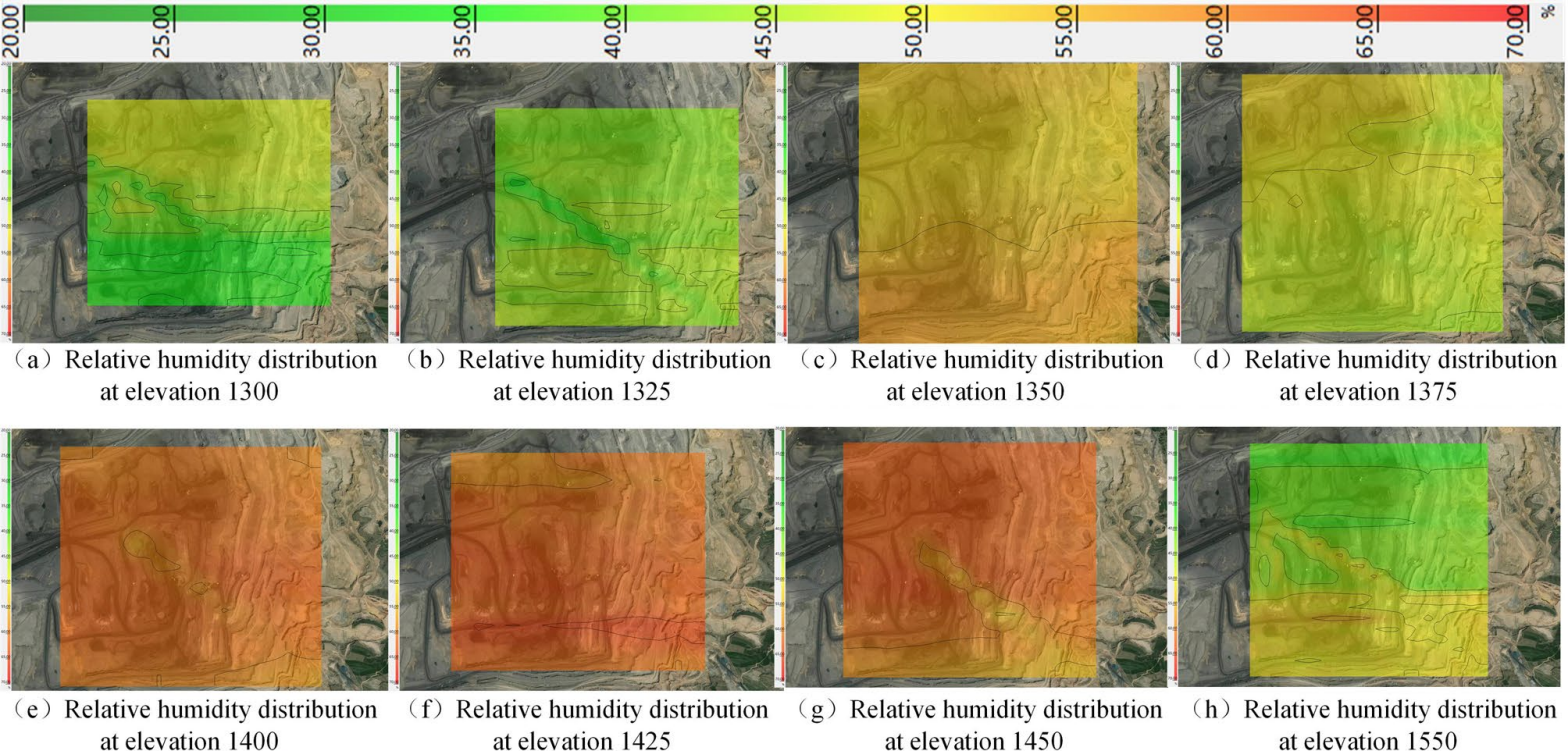
Relative humidity distribution from 1300 to 1550 elevation in winter.

The relative humidity distribution map from 1300 to 1550 elevation was obtained by the kriging valuation method with a threshold value of 20%–70% relative humidity as shown in Fig. 9.

Figure 9 shows that the relative humidity of the north gang is about 37% higher than the south gang at 1300 elevation. The difference between the relative humidity of the north and south gang is smaller at 1325 elevation due to air convection. The overall relative humidity is high at 1350–1450 elevation. The phenomenon of the south gang is higher than the north gang at 1550 elevation.

**Figure 10:**
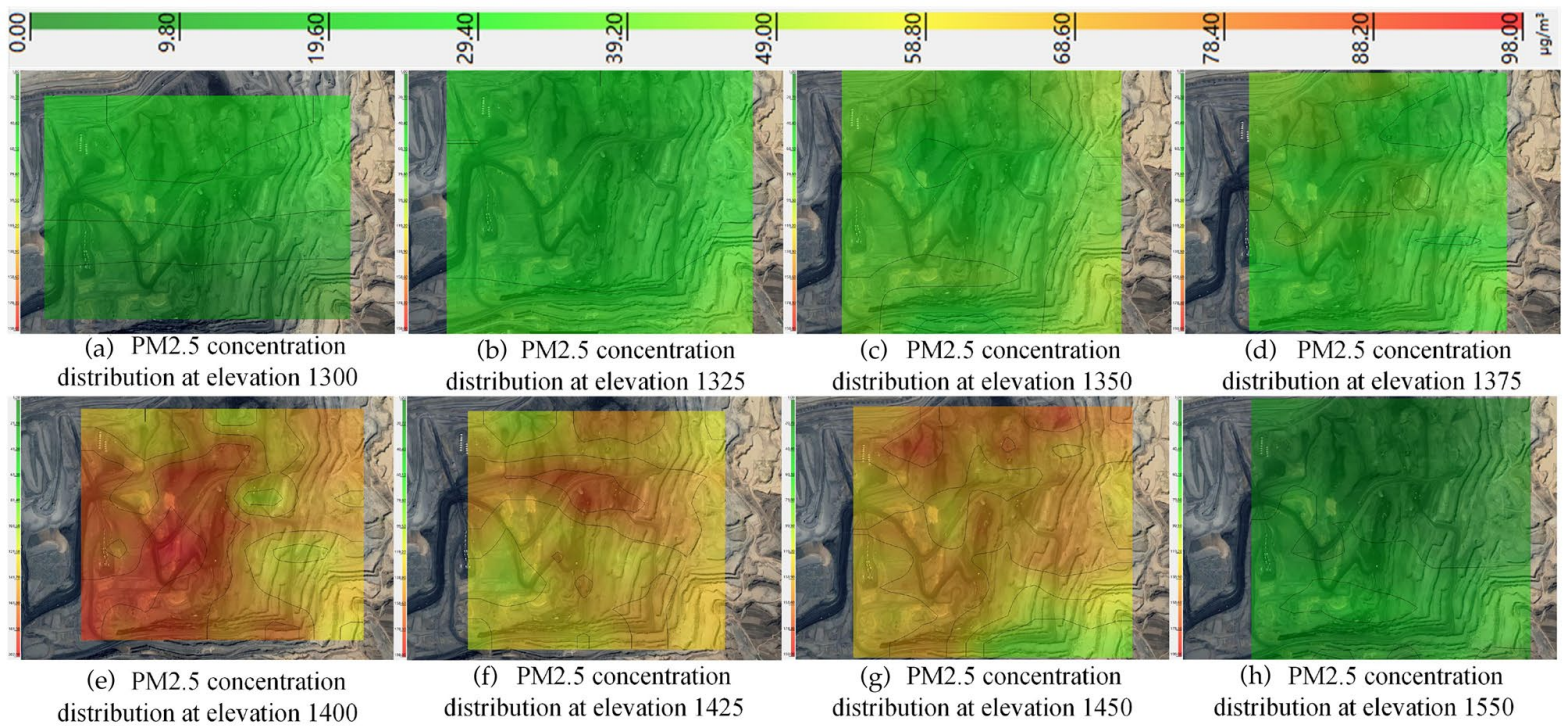
PM2.5 distribution from 1300 to 1550 elevation in winter.

Using the PM2.5 concentration of 198 μg/m^3^ as the threshold, the distribution of PM2.5 concentration from 1300 to 1550 elevation was obtained by the kriging valuation method, as shown in Fig. 10.

As can be seen from Fig. 10, the PM2.5 concentration is high in the north and low in the south at elevation 1300, with an average concentration of 41.50 μg/m^3^. With the diffusion of dust, the PM2.5 concentration difference between the north and south directions from elevation 1325–1375 decreases continuously. With the increase of relative humidity and the influence of the inversion layer, PM2.5 accumulates continuously, resulting in the increase of the average PM2.5 concentration from elevation 1325–1400 from 55.90 μg/m^3^ to 160.40 μg/m^3^. In elevations 1425–1450, PM2.5 concentrations were relatively stable at 133.8 μg/m^3^ and 135.20 μg/m^3^ from elevation 1425–1450. Finally, with the decrease in relative humidity and the destruction of the inversion layer, the PM2.5 concentration decreased to 29.3 μg/m^3^ at elevation 1550, while the PM2.5 showed a north–south divergence trend again, with the concentration in the south being four times higher than that in the north.

**Figure 11:**
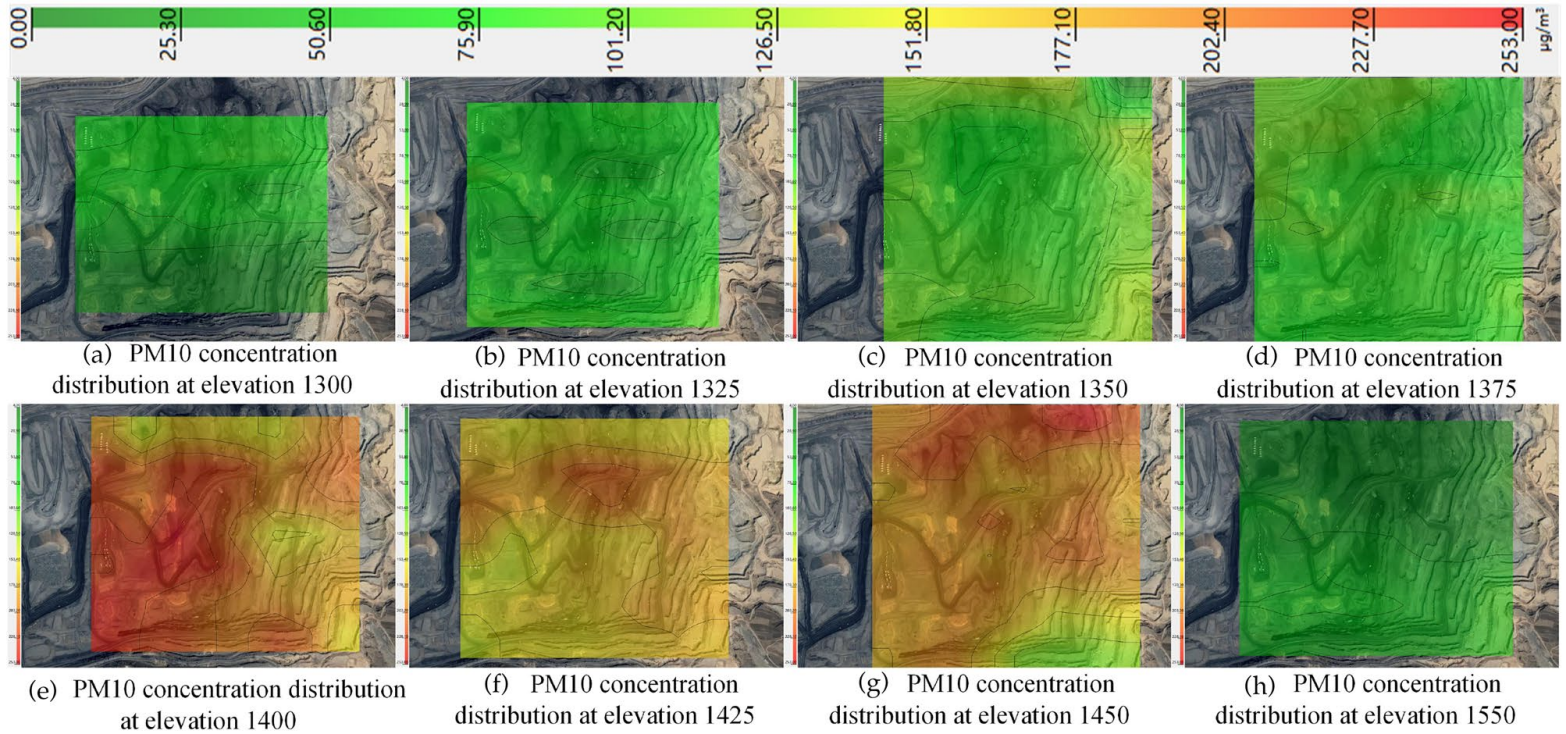
PM10 distribution from 1300 to 1550 elevation in winter.

Using the PM10 concentration of 253 μg/m^3^ as the threshold, the distribution of PM10 concentration from 1300 to 1550 elevation was obtained by the kriging valuation method, as shown in Fig. 11.

As can be seen from Fig. 11, the overall PM10 concentration distribution is similar to that of PM2.5, with the PM10 concentration at elevation 1300 being high in the north and low in the south, with an average concentration of 54.2 μg/m^3^. With the diffusion of dust, the PM10 concentration difference between the north and south directions from 1325 to 1375 elevations decreases continuously. With the increase of relative humidity and the influence of the inversion layer, PM10 accumulates continuously, leading to the increase of the average PM10 concentration from 1325 to 1400 elevations from 73.3 μg/m^3^ to 209.2 μg/m^3^, and the PM10 concentration is relatively stable from 1425 to 1450 elevations, with the concentration The PM10 concentrations were relatively stable at elevations 1425–1450, with concentrations of 175.4 μg/m^3^ and 185.2 μg/m^3^, respectively, and finally decreased to 39.9 μg/m^3^ at elevation 1550 as the relative humidity decreased and the inverse thermosphere was destroyed.

**Figure 12:**
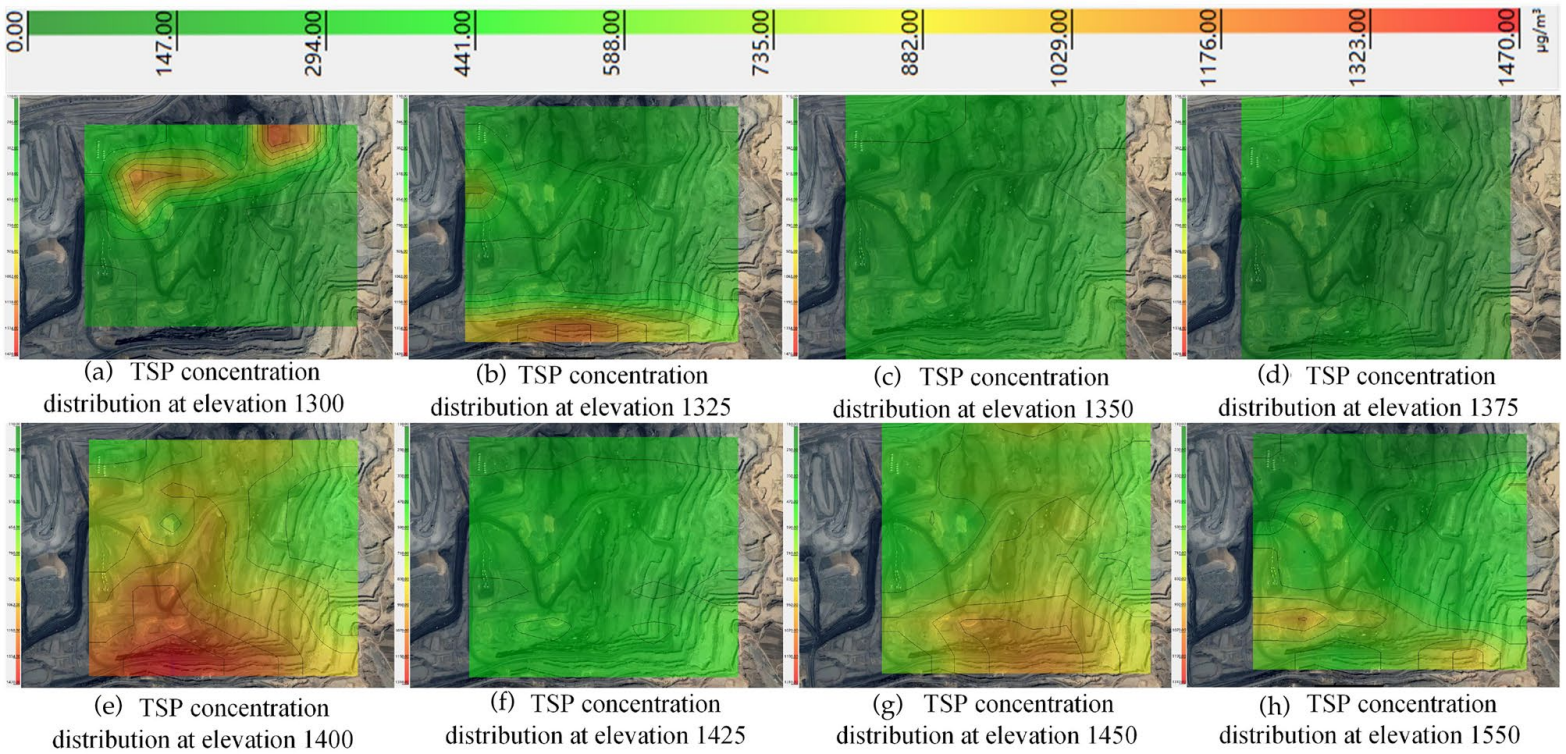
TSP distribution from 1300 to 1550 elevation in winter.

The TSP concentration of 1470 μg/m^3^ was used as the threshold value. The distribution of TSP concentration from 1300 to 1550 elevation was obtained by the kriging valuation method, as shown in Fig. 12.

As can be seen from Fig. 12, the TSP concentration is high in the north and low in the south at elevation 1300, with an average concentration of 371.2 μg/m^3^. With the dust diffusion, the TSP concentration difference between the north and south directions from elevation 1325–1375 decreases continuously. With the increase of relative humidity and the influence of the inversion layer, TSP accumulates continuously, resulting in the increase of the average TSP concentration from elevation 1325–1400 from 573.3 μg/m^3^ to 866.2 μg/m^3^ and the TSP concentration from elevation 1425–1450 increases again with the concentrations of 385.6 μg/m^3^ and 655.1 μg/m^3^. Finally, with the decrease in relative humidity and the destruction of the inversion layer, the TSP concentration decreased to 420.2 μg/m^3^ at elevation 1550.

## Discussion

**Figure 13:**
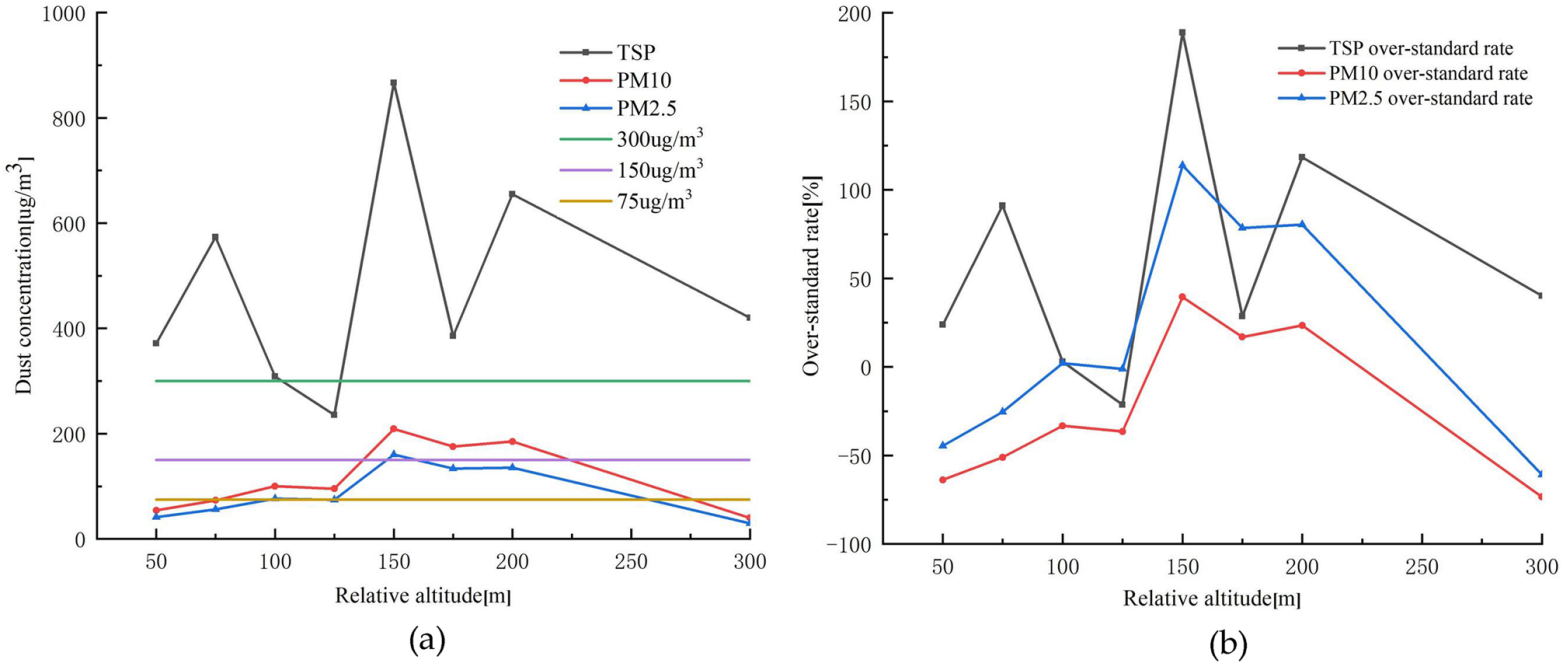
Variation pattern of dust concentration and occupancy rate in winter; (**a**) Dust concentration variation pattern; (**b**) Occupancy rate variation pattern.

The dust distribution pattern was analyzed using the UAV mounted monitoring equipment^23^. The variation of dust concentration in winter was plotted according to PM2.5, PM10, and TSP concentrations and occupancy rates at different elevations, as shown in Fig. 13.

From Fig. 13a, it can be seen that the dust concentration in the open pit mine is mainly concentrated at the height of 150–200 m. The main reason is that the overall high temperature of 150–200 m forms an isothermal layer and an inverse temperature layer, which hinders the diffusion of dust below to the top of the open pit mine, while in the relative humidity range of 25%–50%, the dust concentration increases with relative humidity, thus also causing dust accumulation. Figure 13b shows that the overall exceedance is most serious for TSP, followed by PM2.5. However, at 300 m height, the dust concentrations are all lower than the national standard, indicating that the major impact of dust in the open pit is at 200 m height, while the most serious exceedance is at 150 m height, with TSP, PM10, and PM2.5 accounting for 188.8%, 139.5%, and 113.8%.

Meanwhile, TongWu et al.^27^ explored the microscopic transport and macroscopic diffusion of dust particles in the mine area by numerical simulation methods, and obtained the distribution law of dust particles and the corresponding transport paths in the steady state within the mine area. The results show that the increase of dust particle diffusion velocity is inversely proportional to the particle size, and the larger the particle size in the mine pit, the lower the escape rate of dust particles. It is consistent with the pattern that PM2.5 is mainly concentrated in the 1400–1450 elevation, PM10 is mainly concentrated in the 1400–1450 elevation, and TSP is mainly concentrated in the 1300–1450 elevation in the height direction of field monitoring. It illustrates the reliability of the monitoring.

**Figure 14:**
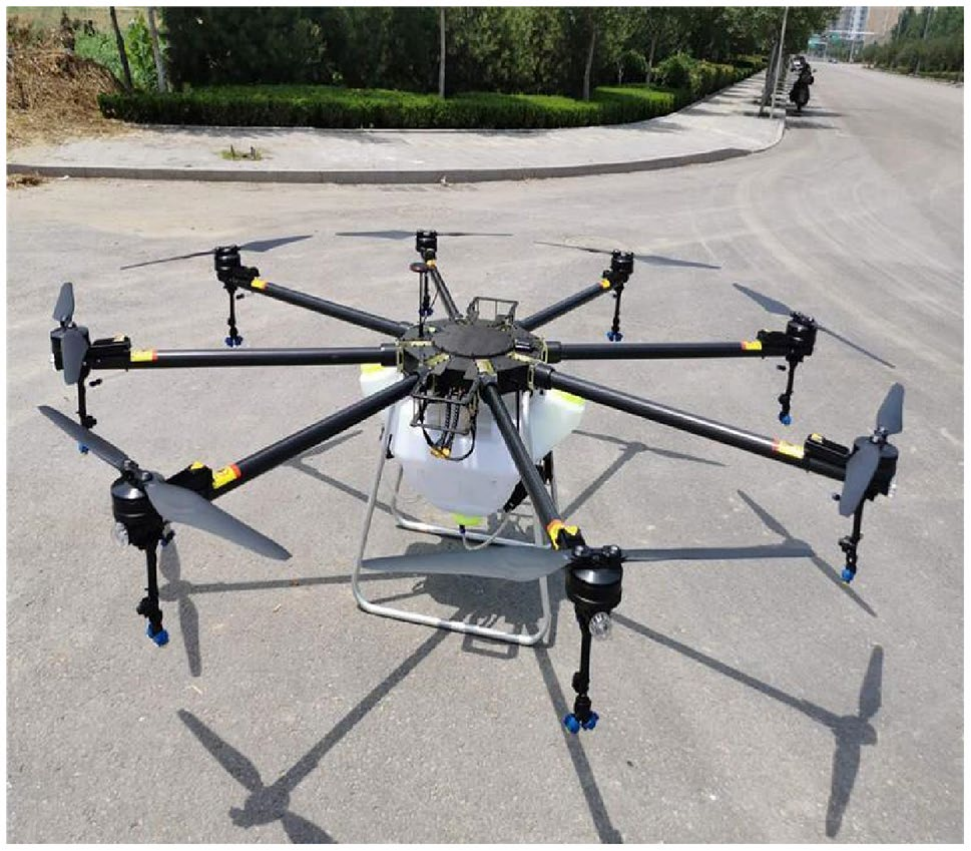
Sketch of the UAV and spraying system.

After obtaining the dust law and then guiding the dust reduction work^28^, with the advancement of UAV technology, this paper proposes to use UAV to carry out dust reduction, which is an effective combination of UAV body and on-board sprinkler device, and then carry out dust reduction work above the open pit mine through remote control of UAV and sprinkler system. The schematic diagram of the UAV and the sprinkler system is shown in Fig. 14.

According to the site monitoring winter isothermal layer and inverse temperature layer are mainly concentrated in the 1350–1450 elevation, so consider spraying in the 1350–1450 elevation to destroy the isothermal layer and inverse temperature layer, enhance air circulation and reduce dust pollution in the open pit mine.

## Conclusion

1. By monitoring the vertical direction of the UAV, the temperature changes in the morning in winter are small. The temperature changes at noon are large, while the isothermal layer gets thinner and thinner as the temperature rises and is constantly being destroyed, which is easy for dust diffusion. The relative humidity is high and varies greatly in the morning. As the temperature rises, the relative humidity decreases and varies little at noon, while the relative humidity is between 40% and 50%, which is not conducive to dust diffusion and deposition, causing the dust to collect in the morning. The dust concentration is high, and the vertical decreasing rate is fast at 10:30 a.m.–11:00 a.m.. The dust concentration is low, and the vertical decreasing rate is small at 11:30 a.m. and 13:00 p.m..
2. A dust distribution analysis method based on the UAV monitoring technology was established. The dust concentration in the horizontal direction of the open pit mine in winter mainly showed polarization at 1300 elevation and 1550 elevation. The PM2.5 in the height direction mainly concentrated at 1400–1450 elevation, PM10 mainly concentrated at 1400–1450 elevation, and TSP mainly concentrated at 1300–1450 elevation. This indicates that large dust particles are easy to settle.
3. The dust concentration in the Anjialing open pit mine exceeds the standard most seriously at elevation 1400, with TSP, PM10, and PM2.5 accounting for 188.8%, 139.5% and 113.8% of the standard, respectively. The main reason is that the overall high temperature at 1350–1450 elevation forms an isothermal layer and inverse temperature layer, which hinders the dust below from diffusing above the open pit. At the same time, in the relative humidity range of 25%-50%, the dust concentration increases with relative humidity, thus also causing dust accumulation. It is suggested that spraying at 1350–1450 elevation destroys the isothermal and inverse temperature layers to enhance air circulation and reduce dust pollution in the open pit mine.

## Data Availability

The data used to support the findings of this study are available from the corresponding author upon request.
